# Loss of calcitonin gene-related peptide (αCGRP) and use of a vestibular challenge highlight balance deficiencies in aging mice

**DOI:** 10.1371/journal.pone.0303801

**Published:** 2024-06-12

**Authors:** Shafaqat M. Rahman, Catherine Hauser, Anne E. Luebke

**Affiliations:** 1 Department of Biomedical Engineering, University of Rochester, Rochester, NY, United States of America; 2 Department of Neuroscience, Del Monte Institute of Neuroscience, University of Rochester Medical Center, Rochester, NY, United States of America; Universite de Caen Normandie, FRANCE

## Abstract

Aging impacts the vestibular system and contributes to imbalance. In fact, imbalance precedes changes in cognition in the elderly. However, research is limited in assessing aging mouse models that are deficient in crucial neuromodulators like Calcitonin Gene-Related Peptide (CGRP). We studied the loss of CGRP and its effects in the aging mouse, namely its effect on both static and dynamic imbalances. Postural sway and rotarod testing were performed before and after a vestibular challenge (VC) in the 129S wild type and the αCGRP (-/-) null mice. Four age groups were tested that correspond to young adulthood, late adulthood, middle age, and senescence in humans. Our results suggest wild type mice experience a decline in rotarod ability due to aging after they reach their prime performance at 6–10 months of age, while the αCGRP (-/-) null mice perform poorly on rotarod early in life but improve with age as they get older, potentially due to vestibular compensation. Our postural sway study suggests that a vestibular challenge can lead to significantly reduced CoP ellipse areas (freezing behaviors) in older mice, and this change occurs earlier in the αCGRP (-/-) null but requires future studies to evaluate anxiety effects. These results indicate that αCGRP is an important component of proper balance and that the loss of αCGRP can contribute to balance complications that may compound with aging.

## Introduction

Aging impacts the vestibular system, and contributes to balance problems and an increased risk of falls [1]. It is estimated that 35% of adults aged 40 and over will experience some form of vestibular impairment, and 85% of people aged 60 and over will experience indications of balance dysfunction [2]. Balance dysfunction causes a nearly 3-fold increase in the odds of falling, and this clinical outcome is highly prevalent in the elderly, affecting 1 in 3 adults aged over 65 per year [3, 4]. In fact, Leach et al. found a linear relationship between day-to-day variability in postural sway and cognitive status in older adults [5], highlighting the deficits that can occur with aging [6–10].

Current knowledge of aging’s effects on human gait and postural control provides context for preclinical researchers to study vestibular dysfunction and aging in mouse models. Mice with loss of calcitonin gene-related peptide (αCGRP-KO) show notable changes in vestibular sensory processing and behavior. The αCGRP-KO mice exhibit a 50% gain reduction in the vestibulo-ocular reflex, shorter vestibular sensory-evoked potentials, and deficits in rotarod ability [11, 12]. Yet, it is unclear if αCGRP loss compounds with aging effects during static and dynamic balance conditions. We hypothesized that αCGRP-KO mice would exhibit severe imbalance phenotypes as a function of aging compared to WT and that a vestibular challenge would aggravate these phenotypes.

We assessed mouse postural sway and rotarod ability on a modified dowel as surrogate behaviors for static and dynamic imbalance in the αCGRP-KO mice and their wild type of complement. Mice were studied across four age groups: young adulthood (2.3 to 6 months), late adulthood (6 to 10 months), middle age (10 to 15 months) and near senescence (15 to 18 months). These age groups correspond to 20 to 30 years, 30 to 40 years, 40–50 years, and 50–70 years in humans [13]. In this study, vestibular challenges (orbital) were used to perturb mouse behavior during static and dynamic conditions.

## Methods

### Animals

The αCGRP-KO and wildtype (WT) transgenic mice were produced and characterized on a pure 129SvEv background obtained from Emeson laboratory [14]. Mice were shipped to the Luebke laboratory as heterozygous (+/-) and genotyped using previously established protocols. The αCGRP heterozygous (+/-) were bred to generate WT (+/+) and homozygous αCGRP (-/-) null used in these studies. The αCGRP (-/-) null strain (referred to as αCGRP-KO) has a targeted deletion of αCGRP due to tissue-specific alternative splicing of the calcitonin/αCGRP gene [14]. Mice were housed under a 12 to 12 day/night cycle at the University of Rochester under the care of the University Committee on Animal Resources (UCAR). A total of 231 mice ‐ 106 WT (45M/61F) and 125 αCGRP-KO (59M/66F) ‐ were used for these studies. A table is provided to indicate the number of mice used across the factors of sex, age, and strain, with many but not all mice repeatedly tested as they aged (**Table 1**). Mice were equilibrated in the test room controlled for an ambient temperature between 22–23° C for at least 30 minutes prior to testing and remained in this room until the experiment was completed. All experiments were approved by the University of Rochester’s IACUC committee and once all testing was completed, mice were euthanized by University of Rochester staff under approved AALAC /NIH approved procedures, which involved exposure to a CO_2_ inhalation chamber followed by secondary physical method. All the behavioral experiments did not require anesthesia nor were not pain inducing so no medications were used as anesthetics as analgesics.

**Table 1:** Sample sizes of 129SvEv WT and αCGRP (-/-) null mice are depicted per age group for rotarod and center of pressure tests. Mice were tested in the range of 2.3–18 months (many were repeatedly tested to conserve overall animal count) and were categorized into four different age groups: early adulthood, late adulthood, middle age, and senescence. The equivalent age in humans is italicized.

Age Groups	Sex	Rotarod—Wildtype	Rotarod– αCGRP KO	Center of Pressure - Wildtype	Center of Pressure - αCGRP KO
2.3–6 months	M	N = 13	13	6	4
early adulthood: *20–30 years*	F	15	12	8	4
6–10 months	M	*11*	10	4	8
late adulthood: *30–40 years*	F	*9*	10	8	9
10–15 months	M	10	11	10	8
middle age: *40–50 years*	F	9	11	16	8
15–18 months	M	9	12	11	10
senescence: *50–70 years*	F	8	13	9	14

### Vestibular challenge–Orbital rotation

During postural sway testing, mice experienced a vestibular challenge (VC) in the form of a steady, orbital rotation at 125 rpm for five minutes (orbital radius = 2 cm). The mice were placed in an acrylic box attached to an orbital shaker’s surface during rotation, and the mouse’s head is fixed in one direction and does not rotate with the axis. The mouse’s body is not fixed but is observed to be stationary during testing, and the head follows an elliptical path. This vestibular challenge is used in another study published from our group [15], and so we continued to use this stimulus for consistency. From our initial studies, we did not see rotarod deficits using the same orbital rotation used in sway experiments. We instead used an orbital rotation but with a greater eccentric rotation then used in sway testing. Thus, for rotarod testing in this study, mice were challenged with orbital rotation, which involves rotating mice at 125 rpm at 2.0 cm from the rotational axis. The mouse’s head is oriented perpendicular to the axis of rotation, so that a different stimulation is imposed on the left versus right semicircular canal because there is a difference in distance from the rotation center to the left versus the right ear. Mice commonly exhibit a transient (1–2 second) body tremor after experiencing the eccentric orbital rotation that is not seen with the less eccentric orbital rotation. This VC is only performed for 30 seconds.

### Rotarod testing for dynamic balance assessment

**Fig 1:**
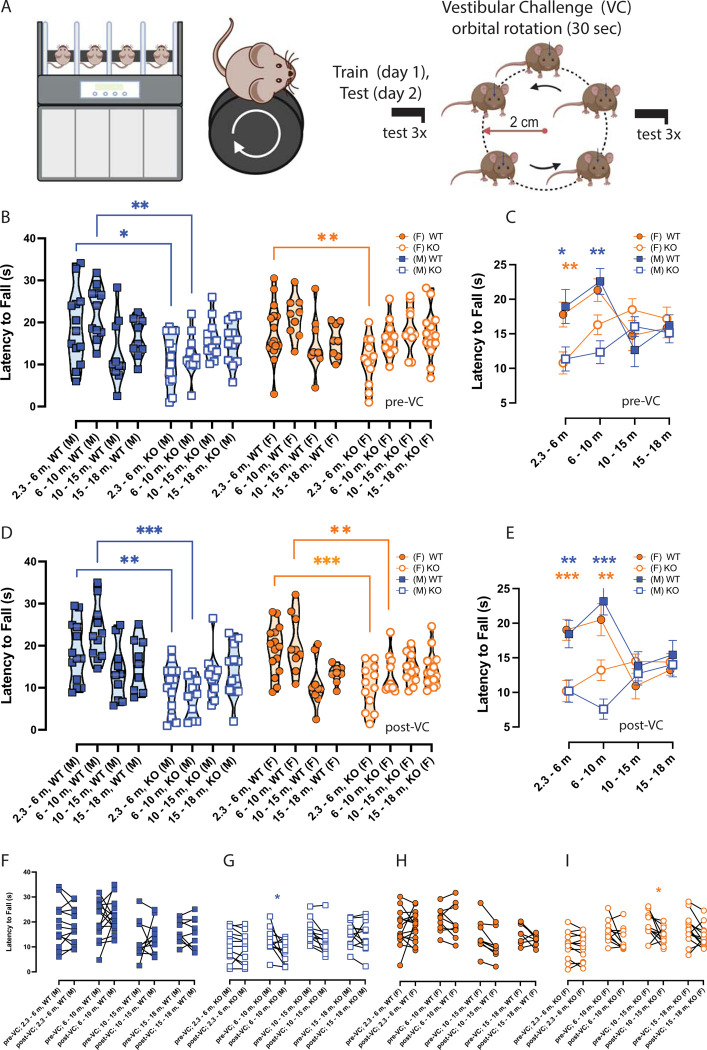
Differences in rotarod performance due to aging, vestibular challenge (VC), and αCGRP loss. **A.** Mice were tested on rotarod configured with rat dowel (r = 3.5 cm) and were assessed for 3 trials before (pre-VC) and after (post-VC) a 30-second eccentric rotation (r = 2 cm) across four age groups. Symbols are depicted as follows: female (F)–orange circle, male (M)–blue square, Wildtype (WT)–closed, αCGRP-K0 –open. αCGRP-KO mice are referred to as KO. **C, E.** Individual MAX LTFs were grouped and depicted as mean ± SEM per age group. **B.** During pre-VC rotarod, WT(M) outperformed KO (M) by 7.6 ± 2.4 s in early adulthood (*p = 0*.*01)* and by 10.3 ± 2.6 s (*p = 0*.*001*) in late adulthood. WT(F) outperformed KO (F) null in early adulthood by 7.0 ± 2.3 s (*p = 0*.*0009*) and appeared to outperform in late adulthood by 5.03 ± 2.8 s, but this was not significant. WT performs similarly to KO on rotarod after 10 months. **E**. Similar differences with age were seen during post-VC rotarod. In either sex, WT outperforms KO in early and late adulthood but no differences were observed after 10 months. **F-I.** Before-after plots were constructed that highlight the effects of the VC on rotarod, but VC’s effects were generally unclear. Full list of ANOVAs and F-values can be found in S1 and S2 Tables. P-values and asterisks are listed as follow: p < 0.05, **p < 0.01, ***p < 0.001, ****p < 0.0001. Blue asterisks compare WT male versus αCGRP-KO male, and orange asterisks compare WT female to αCGRP-KO female. The schematics were created in Biorender.com.

Dynamic balance was assessed with a rotarod (Columbus Instruments) configured with a rat dowel (radius = 3.5 cm). The larger-sized rat dowel was incorporated to facilitate walking and minimize gripping and trapezing behaviors. The rotarod is designed to test four mice simultaneously, and mice were tasked to maintain balance on the rat dowel rotating from 5 to 44 rpm at an acceleration step of 2.4 rpm every 4 seconds. Latency to fall (LTF) is measured when mice fall from the dowel. Two days of rotarod testing were performed. The first day was a training day where mice were tested for 6 to 8 trials. On the second day, mice were briefly trained for 4 trials and were then given a 15-minute rest. After the rest, mice were tested for 3 trials (pre-VC) and were then stimulated with eccentric rotation as the vestibular challenge (see *Vestibular Challenge–orbital rotation* for methods). Mice were then immediately tested for 3 trials on the rotarod after the challenge (post-VC). Approximately 10–30 seconds pass in between subsequent trials during pre-VC and post-VC tests. A schematic of the rotarod methods is shown in **Fig 1A.** All mice, WT and αCGRP(-/-) null, are trained on rotarod using the same training paradigm on day 1 and day 2 to control for learning effects.

### Postural Sway testing for static balance assessment

**Fig 2:**
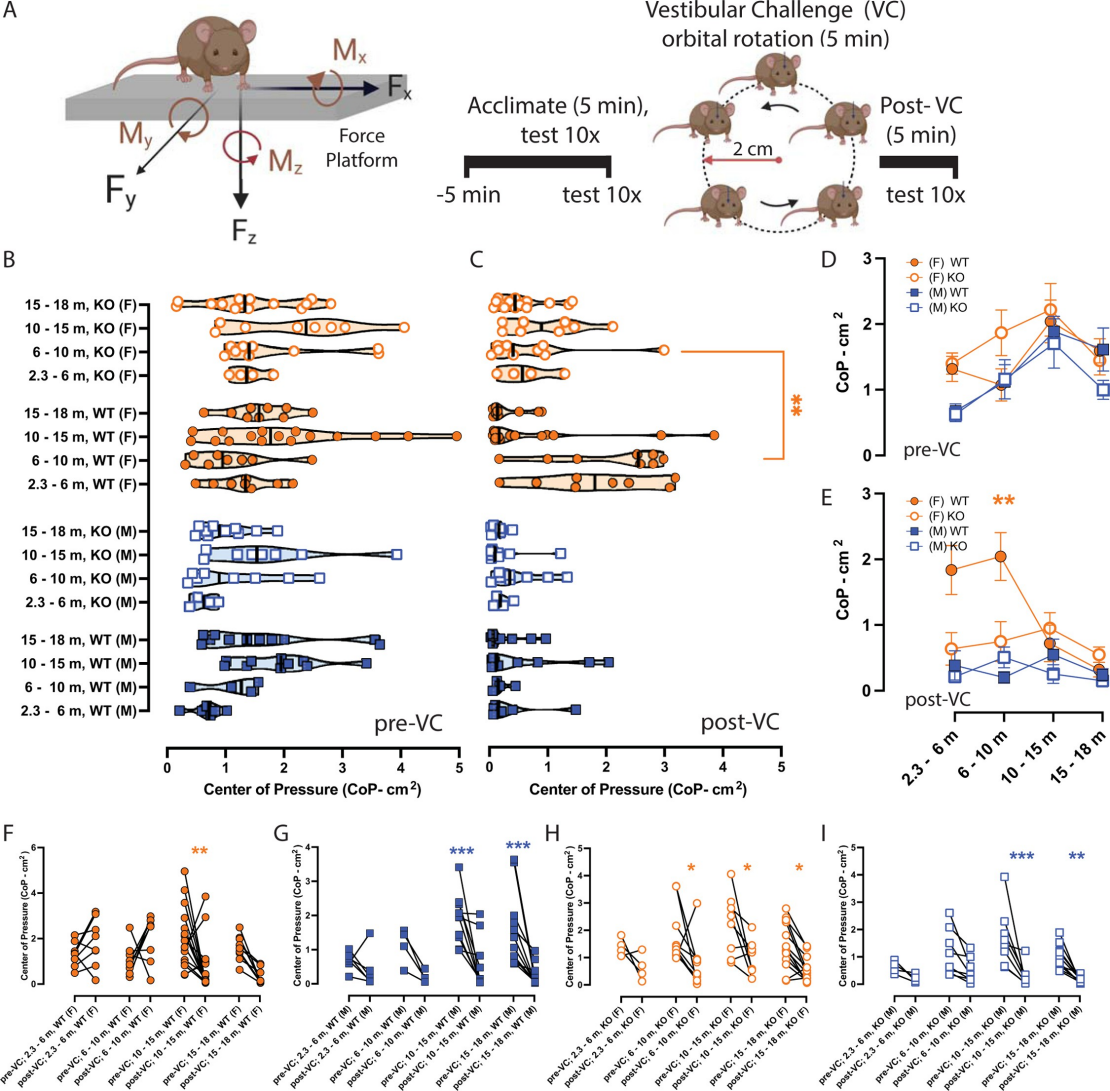
Postural sway (center of pressure–CoP) changes due to aging, vestibular challenge (VC), and αCGRP loss. **A.** AMTI Biomechanics Force platform (model HEX6x6) and its corresponding AMTI automated acquisition software were used to measure 95% confidence CoP ellipses (cm^2^), and the experimental timeline involves an acclimation period (5 minutes) followed by the experiment where 10 trials are captured before and after a 5-minute orbital rotation as the VC. Symbols and color scheme are depicted as follows: female (F)–orange circle, male (M)–blue square, wildtype (WT)–closed, αCGRP-KO–open. αCGRP-KO are referred to as KO. **D-E.** Individual average CoPs were grouped and depicted as mean ± SEM per age group. **D.** During pre-VC, we observed a general increase in CoP area when examining CoP progressing from early adulthood to middle age, but differences were not seen between strains. **E.** For post-VC, WT (F) had larger CoPs compared to KO(F) in early adulthood (*2*.*3–6 m*: *t (p = 0*.*09)*) and in late adulthood (*6–10 m*: *p = 0*.*009)*, however, this difference is gone after 10 months. **F-G**. Middle-aged and senescent WT(F) and WT M) exhibit reduced CoP ellipses due to the VC. **H-I**. KO (F) and KO(M) exhibit reduced CoP ellipses due to the VC from late adulthood to senescence, highlighting a VC sensitivity in KO that arises earlier in age than their WT complement. See bottom halves of S1 and S2 Tables for F-statistics and p-values. The schematics were adapted from “Mouse (anterior 1)” by Biorender.com (2024). Retrieved from https://app.biorender.com/biorender-templates. Blue asterisks compare WT male versus αCGRP-KO male, and orange asterisks compare WT female to αCGRP-KO female. P-values and asterisks are listed as follow: p < 0.05, **p < 0.01, ***p < 0.001, ****p < 0.0001.

The CoP assay is used to measure mouse postural sway as a surrogate for static balance changes [16]. The mice were weighed and placed on a force plate designed to measure the forces due to postural changes in X, Y, Z, and its angular moments in the XY, YZ, and XZ directions (**Fig 2A**). We used the AMTI Biomechanics Force platform (model HEX6x6) and its corresponding AMTI automated acquisition software. An accessory plexiglass cover is placed over the force plate to prevent mice from moving off the force plate. When placed on the force plate, mice are given 2 to 5 minutes to acclimate to the novel environment and minimize their exploratory behavior. After acclimation, 10 trials of the mouse’s CoP area during the pre-VC test were measured by the relative output of four vertical sensors (resolution per CoP trial = 300 samples per second). These trials were captured when the mouse showed no active exploratory behavior (e.g., grooming, standing) and its four paws were touching the surface of the force plate. The mice were then subjected to VC (orbital rotation for 5 minutes) and were then placed back onto the platform. Five minutes after the VC, an additional 10 measurements were measured. A modified MATLAB code was used to analyze CoP data, generating a 95% confidence ellipse that enclosed 95% of the CoP trajectory values computed in a single trial.

### Statistics

Analyses were conducted in GraphPad Prism 9.5. A 10% ROUT removal was used to remove outliers in each mouse’s test prior to calculating its average CoP. In addition, at least six different 95% ellipse areas were used to compute the average. Then, the CoP average (cm^2^) per mouse were further averaged as a mean group CoP ± SE. For rotarod, data was analyzed by first determining the MAX latency to fall (LTF) from the three trials assessed per testing condition. The MAX LTF value(s) were grouped and computed for an average maximum LTF ± SE. Repeated measure ANOVA (RM-ANOVA) and Bonferroni or Tukey post-hoc analyses were the primary statistical tools, depending on which factors were compared from the generated RM-ANOVAs. XY Curves were used to portray meaningful trends in rotarod ability and sway across age groups for either sex or strain. To supplement these trends, we showed violin plots to show individual animal responses and provide insight on the variability in these readouts based on sex, age, and strain. Before-after plots were used to show changes in behavior pre and post a vestibular challenge (VC). Significance is established at p < 0.05, and p-values and asterisks are listed as follow: p < 0.05, **p < 0.01, ***p < 0.001, ****p < 0.0001.

## Results

### Aging and αCGRP loss (-/-) on rotarod

Prior to the vestibular challenge (pre-VC), WT and αCGRP-KO (referred to as KO in figures and statistical comparisons) were trained and later assessed for baseline rotarod ability. Two-way RM-ANOVAs assessed the factors αCGRP loss and aging. F-statistics and p-values can be found in **S2 Table**. Unlike WT, the αCGRP-KO mice tested at early adulthood had poor rotarod ability. In **Fig 1C**, we simplified the violin plot in **Fig 1B** by averaging the MAX LTFs per age for each sex and strain. Bonferroni multiple comparisons tests indicate that WT males outperform αCGRP-KO males by 7.6 ± 2.4 s in early adulthood (*adj*. *p = 0*.*01*) and by 10.3 ± 2.6 s (*p = 0*.*001*) in late adulthood (**left of Fig 1B**). Similarly, WT females outperformed αCGRP-KO females in early adulthood by 7.0 ±2.3 s (*p = 0*.*0009*) and in late adulthood by 5.03 ± 2.8 s, though not significant (**right of Fig 1B**). While WT mice outperform the αCGRP-KO during the first 10 months of age, their rotarod ability declines after 10 months and their performance resemble their similarly aged αCGRP-KO peers.

Using Tukey’s multiple comparisons test, we also assessed the differences between age groups within a particular strain and sex. In WT males, a significant difference was observed between 6–10 m and 10–15 m (*adj*. *p < 0*.*001*) and a trend to significance was observed between 2.3–6 m vs 10–15 m (*adj*. *p = 0*.*07*), though we did not observe a statistical difference between the youngest and oldest age groups in WT males (2.3–6 m vs 15–18 m). In WT females, no significant differences were observed between age groups except for a trend to significance when comparing 6–10 m vs 10–15 m (*p = 0*.*09)*. In both WT males and females, a drop in WT rotarod performance was observed after mice achieve their peak performance at 6 to 10 months of age. In comparison to WT, no significance differences were seen between age groups in αCGRP-KO males even though we observe a steady increase in rotarod performance by 4.69 ± 2.47 s from 2.3–6 m vs 15–18 m, which is the largest increase when comparing age groups within αCGRP-KO males. In αCGRP-KO females, we observed significant differences when comparing 2.3–6 m vs 10–15 m (*adj*. *p < 0*.*01*) and 2.3–6 m vs 15–18 m (*adj*. *p < 0*.*04)*. Like αCGRP-KO males, we also observe a strong increase in rotarod performance in αCGRP-KO females by about 6.40 ± 2.41 s when comparing 2.3–6 m vs 15–18 m, and by 7.69 ± 2.51 s when comparing 2.3–6 m vs 10–15 m.

### VC’s effects on rotarod

Mice were challenged by a 30-second eccentric rotation (see methods) and were immediately tested afterward on rotarod. Post-VC outcomes resembled pre-VC in either sex or strain (**Fig 1D**). In females and males, WT mice outperform αCGRP-KO early and late adulthood but resemble αCGRP-KO performance after 10 months of age (WT vs KO (2.3–6 m): *p = 0*.*004* for males and *p < 0*.*001* for females; WT vs KO (6–10 m): *p < 0*.*001* for males and *p = 0*.*009* for females) (**Fig 1E**). To observe the effects of VC more clearly on an individual animal’s performance, before-after plots were constructed for WT male (**Fig 1F**), αCGRP-KO male **(Fig 1G)**, WT female **(Fig 1H)**, and αCGRP-KO female **(Fig 1I)**. Separate 2-way RM-ANOVAs were used to assess VC’s effects and aging, but the VC did not appear to impact performance, except for the following, particular cases: αCGRP-KO males at 10–15 m showed a decrease of about 4.76 ± 1.50 s due to the VC (adj. *p = 0*.*01*) and αCGRP-KO females at 6–10 m showed a decrease of 3.99 ± 1.52 s (*p < 0*.*05*) due to VC (**Fig 1G and 1I**). Based on this data, the effect of a vestibular challenge in bringing out aging deficits in a mouse model for dynamic imbalance is present but is generally unclear and may require further exploration.

Tukey’s multiple comparisons test also evaluated the differences between age groups within a particular strain/sex after the vestibular challenge. In WT males, a significant difference was observed between 6–10 m and 10–15 m (*adj*. *p < 0*.*001*) and between 6–10 m vs 15–18 m (*adj*. *p = 0*.*01*). Just like Pre-VC testing, post-VC showed no statistical difference between the youngest and oldest age groups in WT males (2.3–6 m vs 15–18 m). In WT females during post-VC testing, we observed differences between 2.3–6 m vs 10–15 m (*adj*. *p < 0*.*005)*, 6–10 m vs 10–15 m (adj. p < 0.002), and 6–10 m vs 15–18 m (*adj*. *p < 0*.*04*).

Post-VC comparisons indicate a drop in rotarod performance with aging that is more aggressive than Pre-VC outcomes, but still hint at a reduced rotarod ability in both sexes of WT after they reach their peak performance at 6–10 m of age. In comparison to WT, αCGRP-KO males exhibited an increase in rotarod performance from 6–10 m to 15–18 m by about 6.45 ± 2.42 s, with no other significant comparisons. αCGRP-KO females also showed no significant differences in rotarod ability with age, but still marginally increased in their performance by 4.18 ± 2.26 s from 2.3.– 6 to 15–18 m.

### Aging and αCGRP loss on Postural Sway

In **Fig 2B**, each symbol indicates the average CoP for a particular animal. As similarly done for rotarod, 2-way RM-ANOVAs on CoP were computed across the factors αCGRP loss and aging during pre-VC and post-VC tests, and these analyses were done separately in males and females (**S2 Table)**. Group average CoPs ± SEM are depicted in **Fig 2D**. During the pre-VC test, we observed a general increase in CoP area from early adulthood to middle age, but no significant differences were observed between WT and αCGRP-KO during the pre-VC tests (**Fig 2B and 2D**).

When looking at the effects of aging within a given sex and sex, we observe that CoP ellipse areas increased from 2.3–6 m to 10–15 m of age, and then dropped when mice aged to 15–18 m. In WT males, we see a statistically significant increase in sway by approximately 1.21 ± 0.45 cm2 from 2.3–6 m to 10–15 m (*adj*. *p < 0*.*04*). However, this was the only comparison that yield statistical significance within wildtype males. We did not observe effects of aging internally within WT females, αCGRP-KO males, and females.

### VC’s effects on Postural Sway

Mice were assessed for postural sway as measured by center of pressure (CoP) changes after a 5-minute orbital rotation (see methods). In females only, WT exhibited a trend of having larger CoP ellipses at early adulthood than the female αCGRP-KO group (**Fig 2C***)*. This was also observed at late adulthood between WT females and αCGRP-KO females (*p* = 0.0009). However, this increased CoP observation is gone as the WT females age past 10 months. From this point onward, no differences were observed between strains at middle age and senescence. Separate 2-way RM-ANOVAs assessed VC’s effects and aging on CoP outcomes. For WT female (**Fig 2F**), post-VC CoP ellipse areas were decreased at middle age by 1.32 ± 0.38 cm^2^ (*p = 0*.*006*) and at senescence by 1.27 ± 0.51 cm^2^ (t (*p = 0*.*07*)). A similar result was observed in WT males (**Fig 2G**), as post-VC ellipse areas were decreased at middle age by 1.34 ± 0.31 cm^2^ (*p < 0*.*001*) and at senescence by 1.27 ± 0.29 cm^2^ (*p < 0*.*001*). Interestingly, the αCGRP-KO showed a response to VC earlier than WT. For αCGRP-KO females (**Fig 2H**), post-VC CoP ellipse areas were decreased in late adulthood by 1.12 ± 0.38 cm^2^ (*p = 0*.*02*), at middle age by 1.27 ± 0.40 cm^2^ (*p = 0*.*01*), and by 0.90 ± 0.30 cm^2^ (*p = 0*.*02*). For αCGRP-KO males (**Fig 2I**), post-VC CoP ellipse areas were decreased in late adulthood by 0.65 ± 0.27 cm^2^ (*t (p = 0*.*08)*), at middle age by 1.45 ± 0.266 cm^2^ (*p < 0*.*001*), and by 0.85 ± 0.24 cm^2^ (*p = 0*.*006*). In addition, mice with significantly low CoP ellipse areas during the post-VC test exhibited a “freezing behavior”, characterized by a lack of activity and a fixed gaze. This behavior is distinct from the usual behavior observed by these mice; however, it was not further quantified in this study.

For sway, we did not observe aging effects within WT males, αCGRP-KO males, and αCGRP-KO females after the VC. However, post-VC outcomes in WT females showed higher sway at 2.3–6 m and 6–10 m than when they were older (2.3–6 m vs 10–15 m, *adj*.*p < 0*.*002*; 2.3–6 m vs 15–18 m, *adj*. *p < 0*.*001*; 6–10 m vs 10–15 m (*adj*.*p < 0*.*001*); 6–10 m vs 15–18 m (*adj*.*p < 0*.*001*)).

### Sex differences in Rotarod and Sway across age, sex, and strain

Using the same MANOVA analyses conducted in this study, we used Tukey post-hoc comparisons to evaluate sex-specific differences in rotarod and sway within strain and age groups, before and after the VC. We observed no statistical differences when comparing pre-VC rotarod, post-VC rotarod, and pre-VC sway for WT females versus males and αCGRP-KO females versus males at any age group. However, post-VC, sway emphasized a significant difference between WT male’s vs WT females at 2.3–6 m (*adj*. *p < 0*.*001*) and at 6–10 m (*adj*. *p < 0*.*001*). This difference is due to significantly larger CoP ellipse areas observed in WT females than is observed in WT males at these age groups.

## Discussion

The vestibular system’s ability to maintain balance and coordination deteriorates as a function of aging. Previous findings show aging’s detrimental effects on balance control in humans and rodents can arise from changes in the vestibular endorgans and vestibular nerve [17–20], chemical imbalances in the inner ear [21], and age-related musculoskeletal deterioration [22, 23]. Mouse models provide an opportunity to assess aging’s effects on behavior when an essential neuromodulator like CGRP is removed from birth.

CGRP is present in the inner ear [12], the trigeminal ganglion, the vestibular nuclei of the hindbrain, cerebral cortical vessels [24], and the cerebellum [25]. We have recently shown that systemic CGRP–delivered intraperitoneally ‐ impacts auditory brainstem responses (ABRs) and vestibular sensory evoked potentials (VsEPs) in the C57BL/6J mouse [15], thus providing evidence that systemic CGRP can modulate inner ear afferent signaling to subcortical and cortical structures. Interestingly, the cerebellum has the most CGRP binding sites in the central nervous system [26]. Aging is associated with a reduction in cerebellar Purkinje cells [27]. Immunostaining studies have indicated CGRP’s presence in motor neurons and cerebellar Purkinje cells of rats, and CGRP signaling is hypothesized to exert a role in cerebellar plasticity which may decline with aging [28]. The αCGRP-KO mice are characterized with a reduced efficacy of the vestibulo-ocular reflex [11], an enhanced activation timing of primary vestibular afferents, a lack αCGRP expression in otolith organs, and a poor rotarod ability at an early age [12]. Since CGRP is present in the vestibular nuclei and the cerebellum [29, 30], losing CGRP from birth is hypothesized to strongly affect the upstream pathways to these structures that ultimately integrate vestibular and motor signals for later conscious perception. Thus, we showed an interest in assessing aging’s effects on the balance behavior of αCGRP (-/-) null mice and extend the current, collective knowledge regarding this novel mouse strain.

Rotarod performance is a mouse surrogate for dynamic imbalance. Our results show that wildtype (WT) mice perform better on the rotarod than αCGRP-KO mice from 2.3 to 10 months, but the difference disappears after 10 months. Our results in the WT correlate with a previous report assessing rotarod and balance beam on the aging C57BL/6 mouse tested at one, nine, and thirteen months of age [31]. In this prior study, a reduction in rotarod performance was observed at nine months and longer latencies to traverse a balance beam were measured at nine and thirteen months of age. Comparatively, the αCGRP-KO mice have poor gait and dynamic balance early in life.

Cognitive and motor deficits in mice that arise with aging can be associated with deficits in signaling of neurotransmitters such as acetylcholine and serotonin [32]. Paired with age-related skeletal muscle mass decline [33] and age-related reduction in cerebellar plasticity [34], these effects can explain the rotarod deficits we observed in older wildtype mice. CGRP co-expresses with acetylcholine in motor neurons [35] and enhances the expression of acetylcholine receptors, and CGRP expresses in the thalamus [36] and cerebellum [37]. Poor rotarod ability in the young αCGRP-KO may be due to changes in interactions between CGRP and acetylcholine on motor control or due to lower Insulin-Like Growth Factor-1 levels which causes impaired spatial learning and cognitive impairments [38]. However, in our study, the αCGRP-KO mice modestly improve their rotarod ability as they get older, with age-related improvements seen in rotarod regardless of provocation by a vestibular challenge. The thalamus is an integrative center for multisensory inputs (vestibular, motor, and visual) and recent evidence suggests cerebellar projections from all three cerebellar nuclei reach most thalamic nuclei [39]. The rat thalamus has been shown to experience adaptive plasticity to bilateral labyrinthine loss [40]. While the cerebellum has the most CGRP binding sites in the brain and CGRP loss may significantly impact cerebellar activity, multisensory compensation mediated by the thalamus may result in recalibrated networks that can explain the modest rotarod improvements in the αCGRP -KO as they age.

We did not observe a significant impact of the vestibular challenge (VC) on rotarod performance for either strain. While not shown in this study, we additionally analyzed the post-VC trials individually, and commonly observed that after the VC, the first trial–when mice are placed on the rotarod dowel approximately 30 seconds after stimulation ‐ is typically associated with a lower LTF than the second and third trials tested subsequently after. This is common in all WT and αCGRP-KO mice tested regardless of sex and age. However, mice quickly recover and that is why their MAX LTF, post-VC does not significantly differ from their MAX LTF, pre-VC, which was the main analysis in this study. It is known that an organism’s vestibular system adapts quickly to changes in its environment, and mice can recover from a vestibular challenge almost immediately [31]. This quick recovery provides a rationale as to why we detected no significant impact of the vestibular challenge on max LTFs (a given mouse’s best effort on the rotarod) and highlights the adaptability of the vestibulo-muscular systems in responding to brief but intense vestibular perturbations. The short-term compensation that arises in mice after the vestibular challenge has been shown before in another study evaluating VC effects in rotarod in the C57BL/6 strain [31], and because compensation does not appear compromised due to age or CGRP loss, we did not focus on this phenomenon in this study.

While we did observe the effects of VC on rotarod in αCGRP-KO males at 6–10 m and αCGRP-KO females at 10–15 m, these effects were not further observed in αCGRP-KO males older than 10 months or in αCGRP-KO females older than 15 months. It is hard to establish a role of age or sex on this assay when the later or earlier age groups do not follow in trends. Due to a lack of meaningful trends in rotarod ability after the VC, we found it appropriate to claim that there was no significant impact of the VC on rotarod performance for either strain.

In postural sway testing, we observed WT females exhibited larger CoP sway ellipses in both early and late adulthood than compared to αCGRP-KO females and to WT and αCGRP-KO males. However, this increased CoP observation in WT females is no longer present as mice age past 10 months. In response to the vestibular challenge (VC), mice exhibit a “freezing” behavior characterized by a lack of movement and a fixed gaze, and we attribute this freezing behavior to an anxiety response. Aging, anxiety, and chronic stress are associated with greater difficulty in performing cognitive tasks in everyday life for patients and especially patients with vestibular disorders [41, 42]. However, we have not evaluated anxiety responses in the 129S WT or αCGRP-KO mice before or after the vestibular challenge (VC) in this study. In future studies, we intend to explore elevated plus maze or open field activity in these mice as readouts for anxiety to supplement this current study and better assess this freezing behavior we observe in mice after the VC. We understand that a reduction in CoP confidence ellipses usually reflects better balance and competes with our hypothesis and analysis. In preclinical and clinical literature, postural sway is associated with postural instability. Clinical studies frequently cite that tight CoP ellipse areas corresponds to a functioning vestibular system, and that increased CoP ellipse areas typically correspond to a neurological disorder like Vestibular Migraine [43] or neurodegenerative disease like Parkinson’s [44]. In mouse models, the center of pressure assay was previously used to detect increases in postural sway in mice with increasing doses of harmaline, a tremor inducing agent, that returned to control values after recovery [16]. Stress is also correlated with increased postural sway in young men [45], and a stiffening strategy is observed in response to postural threats to one’s standing posture and involves co-contraction of the lower body muscles [46]. It is unclear in this study if the tight CoP ellipse areas observed in mice post-vestibular challenge are due to a unique coping mechanism employed by quadrupedal species like mice, whereas bipedal species would still exhibit sway. However, this assessment requires additional experimentation of muscle co-contraction and anxiety which is out of this study’s scope.

When evaluating sex-specific differences in behavior, a confounding variable is the effects of menstrual cycle phases on vestibular performance. While limited in number, there is literature to suggest that changes in hormonal levels during menstrual phases may affect inner ear activity and vestibular networks involved in vestibular perception and performance [47]. In example, progesterone and estrogen are found in the inner ear sensors [48], vestibular nerve, and vestibular nuclei [49], and calcitonin gene-related peptide (CGRP) and its receptor are also found in these regions. CGRP and sex hormone receptors are expressed in the trigeminovascular system, and crosstalk between estrogen and CGRP appear to be involved in migraine [50, 51]. Circulating levels of progesterone and estrogen increase fluid retention during the luteal phase of the menstrual cycle and are associated with peripheral vestibular changes [52]. However, these studies are largely correlative and very few clinical studies have been conducted to establish a link between vestibular performance and systemic hormone levels. In the αCGRP-KO mice, αCGRP loss impacts vestibular sensory processing and performance and this may also be confounded by menstrual cycle phase, but we did not study this question. Future studies are required to tease out the crosstalk between CGRP and hormones on vestibular sensory processing, and if there are significant changes with αCGRP loss. However, the likelihood of differences to be observed in vestibular performance or anxiety of 129S WT or αCGRP-KO mice due to menstrual cycle effects is not currently supported by strong evidence. In example, the timing of the menstrual cycle had no effect on anteroposterior sway or optokinetic function in young women [53]. In addition, young women evaluated for vestibulo-ocular reflex (VOR) gain function and symmetry across their menstrual cycle phases showed no evidence of gain changes or asymmetry [54]. In mouse models, the effects of the menstrual cycle on vestibular performance are even less explored and less understood. We have not found any pertinent studies in mice that correlate menstrual cycle with vestibular function and cannot comment on this feature with confidence. There have been attempts to correlate menstrual cycle phases with anxiety in mice, but results are confusing and not uniform. Some studies suggest differences in mouse anxiety between proestrus, estrus, and diestrus phases [55, 56], whereas other studies showed no effect [57].

In this study, we observed a stark difference in rotarod ability between 129S WT and αCGRP-KO mice in the following age groups: 2.3–6 months and 6–10 months. In the future, we will consider delivering CGRP intraperitoneally or intracerebroventricularly into the αCGRP-KO to observe for reversal effects, and to discern if reversal is mediated peripherally or centrally. In contrast, we did not see strong differences in rotarod between strains in the older age groups, nor did we see a strong effect of strain on postural sway outcomes. In these cases, exploring reversal effects are not worthwhile.

CGRP’s role in inflammation, pain transmission, and in evoking sensory hypersensitivities has supported therapeutic efforts to block CGRP signaling for migraine [58] and COVID therapy [59]. Yet others have found CGRP to act as a neuromodulator with neuroprotective effects [60]. Sensorimotor dysfunction either caused by or resulting in αCGRP loss may require an alternate strategy of re-establishing CGRP signaling in peripheral and central multisensory networks and should be evaluated preclinically in models for sensorimotor deficits due to aging or disease.

To conclude, we investigated the association between aging and balance in both sexes of WT and αCGRP-KO mice. We observe that 129 Sv/Ev WT mice exhibit reduced rotarod ability due to aging and that αCGRP-KO exhibit poor rotarod early in age but improve potentially due to vestibular compensation. In sway, we observed that after the vestibular challenge, wildtype females exhibit higher sway than wildtype males and both sexes of αCGRP-KO. In addition, a vestibular challenge can induce tight CoP ellipse areas that may associate with freezing behavior. These findings instill a better understanding of CGRP’s role in rodent balance control during aging.

## Supporting information

S1 Table Separately done in males and female mice, rotarod and postural sway data during pre-vestibular challenge (VC) and post-VC tests were analyzed with two-way repeated measures ANOVA to assess the factors *aging* and *αCGRP loss*. Bonferroni post hoc analyses computed the difference between wildtype and αCGRP KO at each age group. F-values are listed with respect to degrees of freedom (DF_n_, DF_d_) and p-values are listed accordingly.
(DOCX)

S2 Table Analyzes were separately performed in males and females. To determine the impact of a vestibular challenge (VC) on these balance behaviors, rotarod and postural sway data were further analyzed with two-way repeated measures ANOVA to assess the factors *aging* and *VC effects* in wildtype or αCGRP KO data. Bonferroni post hoc analyses computed the differences between pre-VC and post-VC outcomes at each age group. F-values are listed in degrees of freedom (DF_n_, DF_d_) and p-values are given.
(DOCX)
