# Supplementary material for: Loss of calcitonin gene-related peptide (αCGRP) and use of a vestibular challenge highlight balance deficiencies in aging mice
Source: PLoS One. 2024 Jun 12;19(6):e0303801. doi: 10.1371/journal.pone.0303801 (PMC11168652; doi:10.1371/journal.pone.0303801)
Supplement: S1 Table — Bonferroni post hoc analyses computed the difference between wildtype and αCGRP KO at each age group. F-values are listed with respect to degrees of freedom (DFn, DFd) and p-values are listed accordingly. (DOCX) [file pone.0303801.s001.docx]

**Supplementary Table 1**

**S1 Table. Separately done in males and female mice, rotarod and postural sway data during pre-vestibular challenge (VC) and post-VC tests were analyzed with two-way repeated measures ANOVA to assess the factors *aging* and *αCGRP loss*.** Bonferroni post hoc analyses computed the difference between wildtype and αCGRP KO at each age group. F-values are listed with respect to degrees of freedom (DF_n_, DF_d_) and p-values are listed accordingly.
