# Supplementary material for: Loss of calcitonin gene-related peptide (αCGRP) and use of a vestibular challenge highlight balance deficiencies in aging mice
Source: PLoS One. 2024 Jun 12;19(6):e0303801. doi: 10.1371/journal.pone.0303801 (PMC11168652; doi:10.1371/journal.pone.0303801)
Supplement: S2 Table — To determine the impact of a vestibular challenge (VC) on these balance behaviors, rotarod and postural sway data were further analyzed with two-way repeated measures ANOVA to assess the factors aging and VC effects in wildtype or αCGRP KO data. Bonferroni post hoc analyses computed the differences between pre-VC and post-VC outcomes at each age group. F-values are listed in degrees of freedom (DFn, DFd) and p-values are given. (DOCX) [file pone.0303801.s002.docx]

**Supplementary Table 2**

**S2 Table. Analyzes were separately performed in males and females.** To determine the impact of a vestibular challenge (VC) on these balance behaviors, rotarod and postural sway data were further analyzed with two-way repeated measures ANOVA to assess the factors *aging* and *VC effects* in wildtype or αCGRP KO data. Bonferroni post hoc analyses computed the differences between pre-VC and post-VC outcomes at each age group. F-values are listed in degrees of freedom (DF_n_, DF_d_) and p-values are given.
